# The Ubiquitous Use of Polyethylene Glycol in Pharmaceutical Design and Development: Technological Aspects and Future Perspectives

**DOI:** 10.3390/nano15231762

**Published:** 2025-11-24

**Authors:** Iliana Christoforou, Anastasios Kalatzis, Angeliki Siamidi, Marilena Vlachou, Stergios Pispas, Natassa Pippa

**Affiliations:** 1 Section of Pharmaceutical Technology, Department of Pharmacy, School of Health Sciences, National and Kapodistrian University of Athens, Panepistimioupolis Zografou, 15771 Athens, Greece; iliana.christof@gmail.com (I.C.); kalatzistas@gmail.com (A.K.); asiamidi@pharm.uoa.gr (A.S.); vlachou@pharm.uoa.gr (M.V.); 2 Theoretical and Physical Chemistry Institute, National Hellenic Research Foundation, 48 Vassileos Constantinou Avenue, 11635 Athens, Greece; pispas@eie.gr

**Keywords:** PEG, drug delivery, active pharmaceutical ingredient, solid dosage forms, nanocarriers, hydrophilicity, drug release

## Abstract

Polyethylene glycol (PEG) has been extensively utilized in drug formulations due to its multifunctional properties, i.e., hydrophilicity and biocompatibility. The roles played by PEG (as a drug delivery carrier and a solubilizer) improve the dissolution profile of several active pharmaceutical ingredients (APIs), leading to an improved absorption, distribution, metabolism, excretion, and toxicity (ADMET) profile. Moreover, PEG aids in upgrading the existing mechanical properties (as a binding agent, a plasticizer, etc.). Furthermore, PEG, due to its unique ability to provide “stealth” properties, is a valuable tool in pharmaceutical nanotechnology. Exploiting physicochemical variables, PEG acts as a coating/conjugation component of nanocarriers for ameliorating permeability and enhancing in vivo circulation without clearance by the body’s immune system. Additionally, PEG’s presence at the target site decreases external interactions and enhances the pharmacological attributes in terms of loading efficiency and controlled release. Nevertheless, cases of hypersensitivity or allergy, as well as anaphylactic shocks and allergic reactions, have been detected. The topic of this article is the exploitation of PEG’s physicochemical properties in the study of drug delivery, focusing on solid dosage forms and nanovesicles, along with the evaluation of its contribution to the fabrication of safe delivery and theragnostic systems.

## 1. Introduction

**Figure 1:**
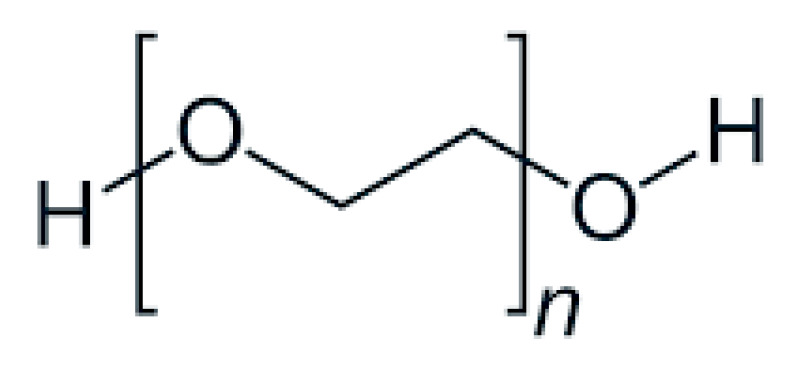
The chemical structure of PEG [5].

Polyethylene glycol (PEG) (Figure 1) is a linear polymer that consists of repeated units of ethylene glycol [-(CH_2_CH_2_O)_n_], with its molecular weight usually ranging from 200 to 20,000 g/mol. Owing to its favorable physicochemical properties, polyethylene glycol (PEG) enhances the stability and sustainability of drug delivery systems, facilitating controlled and efficient transport of active pharmaceutical ingredient (APIs) to the target site. Acquiring flexibility and high hydrophilicity, it acts either as a carrier or a solubilizer for orally administered drugs with reduced recognition from the body’s defense mechanisms and acts as a coating agent of nanoparticles for enhanced permeability and greater targeted release. Characterized by biocompatibility and non-toxicity, PEG represents an eco-friendly alternative in the study of drug delivery [1,2,3,4,5].

The oral administration of PEG-containing drugs (tablets, capsules, etc.) induces drug delivery, achieving localized treatment while offering higher patient compliance, stability, and dose accuracy. The manufacturing process is characterized by low cost, design flexibility, and scalability, although the API has to be dissolved prior to its absorption. PEG’s presence in solid dosage forms leads to the solubilization of Class II or IV oral drugs, affecting their bioavailability and also contributing to the formation of dosage forms created using the solid dispersion method or acting as a carrier in these cases [6,7].

Drug delivery attained by nanotechnology schemes has also been developed using intranasal or intravenous administration of nanovesicles, like liposomes and micelles, loaded with APIs. PEG’s attributes allow for the minimization of any side effects, as well as the fabrication of a chemically stable environment for therapeutic efficacy [1].

This article focuses on five categories of nanocarriers:

- Liposomes: Vesicular structures made of lipids with hydrophobic tails on the inside and hydrophilic heads on the outside, which allows them to capture both hydrophobic and hydrophilic APIs inside their aqueous core [8]. PEG manages to bind onto liposomes by forming PEG–lipid conjugates that get integrated into the liposome bilayer using linkages like amide bonds [9,10].
- Micelles: Spherical or non-spherical structures made of amphiphilic low- or high-molecular-mass molecules with hydrophilic heads and hydrophobic tails. Their nonpolar interior allows them to entrap only hydrophobic APIs [11]. PEG is successfully attached to micelles through amphiphilic block copolymers and surfactants via covalent bonds or lipid-based conjugation [12,13].
- Polymersomes: A class of artificial vesicles formed from synthetic amphiphilic block copolymers. They are able to enclose hydrophilic APIs inside their aqueous core, whereas hydrophobic APIs are enclosed inside the hydrophobic block copolymer’s outer bilayers [14]. PEG requires covalent conjugation to amphiphilic block copolymers during synthesis in order to form morphologically strong structures [15,16].
- Inorganic nanoparticles: A wide variety of nanomaterials that induce conjugation with both hydrophilic and hydrophobic APIs through chemical covalent bonds [17]. PEG utilizes its electrostatic stability to connect with inorganic nanoparticles. The type of bond depends on the type of material used and is correlated to the nanoparticle’s surface topography [18,19].
- Niosomes: Non-ionic surfactants and neutrally charged lipid components. They possess the ability to entrap both hydrophilic and hydrophobic APIs inside their core structures [20]. PEG gets embedded into the niosome bilayer during the formation of the latter via hydrophilic interactions with the surfactants [21,22].

PEG’s physicochemical properties play a substantial role in PEGylated nanocarrier systems. It has been proven that higher PEG molecular weights within the range of 2–20 kDa enhance the steric stabilization of the nanocarriers and prolong the in vivo circulation within the bloodstream. As far as the stereochemistry of PEG is concerned, studies indicate that utilizing a branched structure has a significant effect on the surface density and the immunogenicity of the derived nanosystems in contrast to the linear one. Branched PEG provides a wider weight distribution with greater resistance to the accelerated blood clearance (ABC) phenomenon, thus improving the biocompatibility profile and the pharmacokinetics [23,24,25,26].

Apart from the use of PEG in nanocarriers, the purpose of this work is also to investigate the use of PEG in solid dosage form, a pharmaceutical form that is commonly used in therapy due to the advantages that it has. In more detail, it provides higher patient compliance and the potential of targeted administration, and it is also cost-effective, production- and utilization-flexible, and easily scalable [6,7]. Due to its high hydrophilicity, polyethylene glycol, acting as a solubilizer, solubilizes Class II and IV drugs and improves their bioavailability [3,27,28]. In addition, in another technique (the solid dispersion technique), PEG is used as a carrier or for the formation of the dispersion and improves the dissolution profile of the drug and subsequently its absorption after oral administration due to the fact that PEG increases formulation wettability and reduces drug crystallinity [6,29]. It has also been used as a cosurfactant or as an emulsifier in self-nanoemulsifying tablets [30,31]. Moreover, in coated tablets, it has played the important role of a plasticizer for film formation or of a detackifier for protection against degradation [32].

The topic of this article is the exploitation of PEG’s physicochemical properties in the study of drug delivery, focusing on solid dosage forms, including several subclasses of tablets, capsules, and nanovesicles, along with the evaluation of its contribution to the fabrication of safe and effective pharmaceutical dosage forms. The authors aim to describe the uses of PEG in well-established dosage forms, i.e., tablets, capsules, and liposomes, and innovative delivery platforms, i.e., self-nanoemulsifying, orodispersible, and osmotic pump tablets, as well as PEGylated polymersomes and inorganic nanoparticles, underlining their unique properties to ameliorate the ADMET profile of APIs. In all cases, PEG display specific functions such as solubilization, drug release moderation, and biocompatibility improvement. The authors chose to investigate the case studies of solid dosage forms and nanoparticulate and make comparisons between them about the properties and added value of PEG in the design and development of these formulations. The interconnection between PEG physicochemical characteristics and the added value in the final dosage form are emphasized in an extensive literature review.

## 2. Methods

The literature search was performed under Advanced Search in PubMed. For the solid dosage forms, “Title/Abstract” was selected in the Advanced Search Builder, and PEG was entered as the search term, adding (with AND) either tablets or capsules. For the nanoparticles, “Title/Abstract” was selected in the Advanced Search Builder, and PEGylated was entered as the search term, adding liposomes, micelles, polymersomes, inorganic nanoparticles, niosomes, or nanoparticles. Finally, in both cases, only research papers (Table 1 and Table 2) which were published from 2019 to 2024 were selected.

## 3. Results and Discussion

Firstly, we are going to discuss the role of PEG in solid dosage forms, paying special attention to the relationship between the molecular characteristics and the release profile of the encapsulated API.

### 3.1. Solid Dosage Forms—Tablets

In this section, all the classes of tables will be analyzed in terms of the added value of the presence of PEG.

#### 3.1.1. Self-Nanoemulsifying Tablets

The objective of the research study by Ali et al. was the manufacturing of self-nanoemulsifying chewable tablets of the poorly soluble Tadalafil in order to improve both solubility and bioavailability. For this purpose, PEG-40 as a surfactant and PEG-400 as a cosurfactant, both with high-membrane/tissue-transmittance properties, were used. Based on the results, the C_max_ and AUC of these tablets were 2.33- and 5.33-fold higher than those of directly compressed tablets (DCTs). As for the dissolution percentage, it was 84%, compared with 14% of the DCTs, after 15 min. As a result, the formulation had improved solubility and oral bioavailability, and the release of the drug was immediate. Its solid form facilitated administration and improved both stability and patient compliance [33].

**Figure 2:**
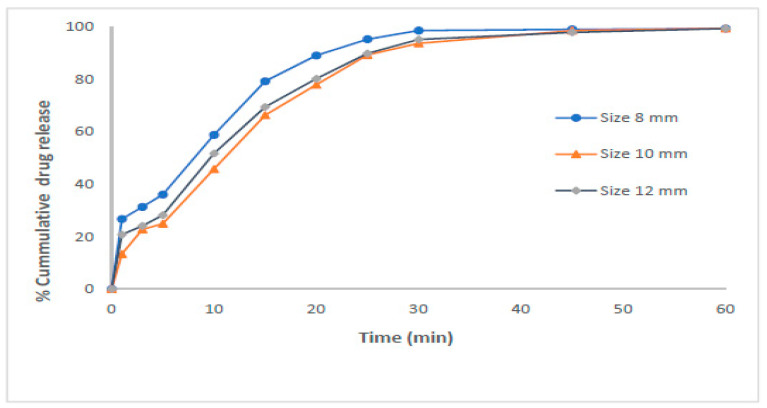
In vitro release profile of Dapagliflozin tablets of varying sizes [34].

In another study with the same goal, which was the development of self-nanoemulsifying tablets of the drug Dapagliflozin propanediol, which has low solubility and permeability, PEG 400 was used as a cosurfactant in the liquid phase and PEG 600 as an excipient in the solid matrix, which also acted as an emulsifier. The researchers used semi-solid extrusion-based 3D printing, and the small droplet size of the tablets, around 100 nm, resulted in improved dissolution and absorption. The latter was due to the high stability of the active substance carrier, the self-nanoemulsifying system. Moreover, the release was immediate, and there was no lag time. In Figure 2 the in vitro release profile of tablets of different sizes is presented [34].

To overcome the solubility problem of Sertraline in a previous study, the authors produced again a self-nanoemulsifying drug delivery system-derived tablet. The development of a tablet form was implemented by using PEG 200 as a cosurfactant and resulted in an increase in bioavailability, since compared with the Sertraline suspension, the relative bioavailability was 386%. The results from Differential Scanning Calorimetry (DSC) studies of the formulation showed that the increased solubility was due to the amorphous state of the drug molecules compared with the initial crystalline state. In addition, the above tablet form improved clinical efficacy [30].

Moreover, using PEG 400 as a cosolvent to form self-nanoemulsifying liquisolid tablets seems to be an effective way to administrate Furosemide, a drug with low solubility and low permeability. A study showed that in phosphate buffer with pH 5.8, 89% of Furosemide was released in 60 min. Both DSC and X-ray diffraction revealed that in the final formulation, the model drug lost its crystalline nature and became amorphous and more soluble. However, at high drug loading, in the case of tablets being highly compressed, there is a risk that they are fragile. This can be solved by reducing the percentage of drug loading, but this will in turn form a tablet with low weight, which is very difficult to swallow [31].

#### 3.1.2. Orodispersible Tablets

Cefdinir is used as an antibiotic against Gram-positive and -negative bacteria. The use of PEG 6000 as a hydrophilic carrier was catalytic for the increase in Cefdinir’s solubility (to around 4.50 mg/mL at pH 1.2), which is generally low at lower pH. Hence, by using meglumine as an alkalizer for the production of the solid dispersion and PEG 6000 in a drug–PEG ratio equal to 1:1, researchers formulated orodispersible tablets (solid dispersion-based). The drug released from these tablets was between 69% and 94% [93].

In another investigation researchers manufactured orodispersible tablets of Metformin hydrochloride and Gibenclamide (poorly soluble) in order to reduce pill burden, as well as overcoming difficulties in swallowing that patients with type 2DM had. In that case, PEG 6000 played the role of a binding agent and also improved the dissolution profile of the tablets. Moreover, it gave adequate mechanical strength to the final formulation. After 30 min, the percentages of released drug were between 89% and 95% for Metformin and between 84% and 91% for Gibenclamide [94].

Orodispersible tablets were also manufactured for the oral administration of Glimepiride, a drug with low solubility, using PEG 5k for the formation of solid dispersions with the purpose to improve the dissolution profile. In detail, PEG 5k enhanced the water solubility, the stability, and also the bioavailability of the drug. However, from the results, it was evident that Polyvinylpyrrolidone (PVP), which was also tested for the preparation of the solid dispersion, increased the solubility of the dispersion more than PEG 5k. The final formulation showed rapid disintegration, since it occurred in 13 s, and released in vitro 90% of Glimepiride after a time of 40 min [95].

#### 3.1.3. Tablets with Fast Dissolution

PEG with a molecular weight of 4000 g/mol was very useful for the preparation of Haloperidol solid dispersion for the formulation of fast-dissolving tablets, with the intention to improve the solubility and, as a result, the dissolution profile of Haloperidol. The preparation of the dispersion was made through either the melting method or the solvent evaporation method, but the first showed better performance, since the dissolution percent of the composition of a Haloperidol/PEG 4000 1:8 mixture was 104 ± 0.23% at 60 min. Furthermore, these tablets seem to be valuable for people that face problems in swallowing conventional tablets, and they also show quick-onset action [35].

**Figure 3:**
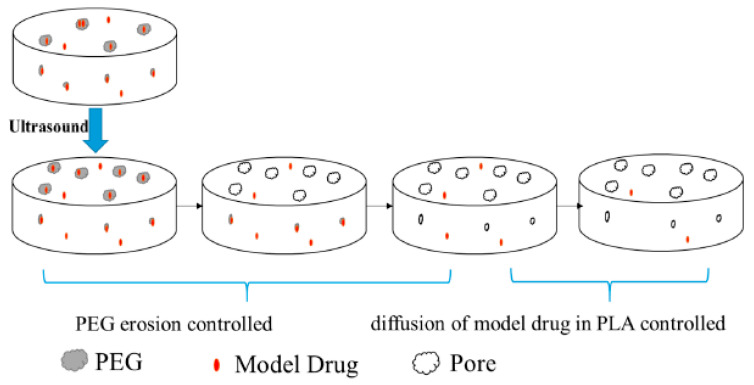
Mechanism of release [96].

The objective of the investigation by Bao et al. was to study the way in which the combination of ultrasound and PEG with a molecular weight of 6000 g/mol affect the release profile of Methylene Blue. Since PEG has high hydrophilicity as well as high dissolution speed, the achieved release rate of the drug was equal to 70%. PEG was part of the carrier but also formed pores. As a result, in the beginning, the rate of dissolution was high, since the release was primarily controlled by erosion, and afterwards, the formulated pores allowed for the controlled release of the drug, since the basic mechanism was diffusion (Figure 3) [96].

#### 3.1.4. Coated Tablets

The purpose of the research study by Kestur et al. was to investigate the effect of using coating excipients in order to protect the active substance Peglitazar, which is prone to both acid and basic degradation and is sensitive to excipients. PEG 3350 had to play an important role, since it was a plasticizer (for optimal film formation) or a detackifier with Hydroxypropyl Methylcellulose (HPMC) or Polyvinyl Alcohol (PVA) as coating material, respectively. The presence of PEG was critical due to the fact that it resulted in improved solubility and reduced degradation of the coated tablets. However, a limitation of the study was that it is necessary to know the mechanism of degradation in order to make a decision on the optimal coating excipient [32].

Another study which had the aim to manufacture a protective film for Herniarin glabra tablets examined the use of PEG with a molecular weight of either 1500 or 4000 g/mol as a plasticizer. The special characteristic of the polyethylene glycol utilized is its insolubility under both acid- and neutral-pH conditions. The performed stability studies showed that the coating process resulted in a final formulation that had physical as well as dissolution stability after storage for 12 months at room temperature and also under accelerated aging conditions. Moreover, the weight gain of the coated tablets was lower than that of the uncoated ones. For example, at 75%RH, the weight gain was 5.7% vs. 16.1%, respectively [36].

The objective of the studies performed by Moutaharrik et al. was the formulation of compact tablets without adding excipients in order to reduce the forces that are applied on them. Hence, they manufactured cushion-coated pellet systems of acetaminophen, using PEG 1500 for the preservation of the integrity of the tablets, the promotion of compaction, and rapid disintegration. This relied on the fact that PEG has high solubility and also low melting temperature, between 44 °C and 48 °C. Hence, the final formulation showed a disintegration time of between 8 and 9.7 min [97].

#### 3.1.5. Osmotic Pump Tablets

Researchers presented a different type of tablet formulation that could overcome the problems of common tablets, namely, their short lifetime and unstable concentration in plasma. In more detail, they fabricated double-layer osmotic pump tablets of Actarit, a drug for rheumatoid arthritis, which was released from this formulation in a prolonged mode. From the results, T_max_ was observed to be 2-fold higher than that of the commonly used tablets. PEG 4000 played a dual role, since it took part in pore formation but was also a plasticizer, and as a result, it increased the toughness and strength of the tablets (as a coating film) [98].

Long-term and controlled release of the substance Lorcaserin hydrochloride was achieved by manufacturing osmotic pump tablets that had, in the coating, a solution of PEG 4000 as a pore former. The cumulative release rate was equal to around 95.7%, and the kinetics were zero-order with a good correlation coefficient. The fabricated formulation had also a constant rate of release for 16 h, and as a result, it had a longer effective concentration. It was the first time in which osmotic pump tablets which released the drug at a constant rate were prepared. This resulted in a drug plasma concentration with fewer fluctuations and reduced side effect probability. Moreover, the osmotic tablets improved patient compliance mainly due to the reduction in the number of tablets to be taken [99].

#### 3.1.6. Liquisolid Compact Tablets

Itraconazole is a drug with low aqueous solubility, which results in a poor bioavailability of around 55%. For the improvement in its bioavailability, researchers used as a non-volatile solvent PEG 600, in which the drug had a solubility of around 3.8 mg/mL. This liquid vehicle led to the design of liquisolid tablets and capsules, with the latter showing higher bioavailability. The relative bioavailability of the tablets compared with the plain drug was equal to 1.87. Moreover, it was very important that the active substance maintained its antifungal activity in the formulated tablets [100].

Shah et al. discovered the use of PEG 400 as a non-volatile solvent, which played an important role in the administration of the poorly soluble Rivaroxaban (5–7 μg/mL), which acts also as a substrate for P-gp. The liquisolid compact tablets that were fabricated accelerated the onset of the action, as the disintegration time was 69 ± 2.46 s, and they also demonstrated 100% viability. Additionally, the P-gp efflux was inhibited due to the presence of PEG. The DSC thermograms showed the change from crystalline-to-molecular dispersion state, and the formulation had enhanced bioavailability. As a result, this can reduce the dose and the side effects and also affects patient compliance positively with tablets [7].

#### 3.1.7. Formulations with Modified Release

The difficulties that could arise during the fabrication of drug formulations with fused deposition technology seem to be associated with the direct extrusion method that manufactures formulations in a one-step-only process. Thus, by using PEG 4000 as a plasticizer, Sánchez-Guirales et al. printed tablets of Nifedipine (for the treatment of hypertension) with a weight of less than 100 g but with high drug load, 25%. Moreover, these tablets released the drug in a sustained way, a feature especially important due to the fact that utilization of immediate-release tablets could lead to hypotension. Less than 30% was released after 4 h, but after 24 h, this percentage reached up to 99.9%. Furthermore, another advantage of this method is that it can print the final formulation within 2 min, and as a result, it gives the opportunity of personalized therapy [101].

Sustained-release tablets were also successfully fabricated using digital light processing 3D printing. In a previous investigation PEG-DA 700, which is photoreactive and has the property of crosslinking under UV radiation, and PEG 400, which has high hydrophilicity and helps dissolution of Ibuprofen, were used. From the results at 405 nm wavelength, the percentage of in vitro drug release achieved after 24 h was 79.00 ± 1.15%. In addition, bioavailability was increased compared with the commercially available treatment, since the C_max_ values were 30.124 μg/mL and 9.715 μg/mL, respectively. However, this method needs to overcome the problem of the limited availability of agents that are used for the formulation [37].

Additionally, the formulation of Atomoxetine hydrochloride tablets with modified release was accomplished via digital light processing and also by using PEG-DA and PEG 400 for the improvement in their dissolution rate and strength. In particular, PEG-DA acted as a photopolymer that, in the presence of light and of a photoinitiator, polymerized and could also overcome the bitter taste of Atomoxetine hydrochloride, which is very important since it is mostly given to children. PEG 400 is an excipient that plays an important role in overcoming release problems. Moreover, this formulation is very promising since it released the model drug after 8 h and the achieved dose range allows for personalization of the therapy [102].

The objective of another study was to develop sustained-release tablets of Divalproex sodium. By using PEG 8000, researchers controlled hygroscopicity in order to produce these tablets even in an environment with high humidity. The technique of solid dispersion and the use of PEG 8000 and HPMC led to a controlled-release profile (which resembles the Korsmeyer–Peppas kinetics model of drug release). This type of release has many advantages, since it decreases the time during which patients need to take their treatment per day, and as a result, patients show better compliance with it. Moreover, the side effects are fewer, but the dose should be increased in order to achieve the same bioavailability as the conventional treatment. Another disadvantage is that researchers could not perform X-ray measurements in order to understand better the molecular interactions [38].

Furthermore, an attempt made in order to develop a safer treatment formulation of Lopinavir combined with Ritonavir for children with HIV led to a tablet with zero-order sustained release after 24 h. The work is very important because there are availability problems with commercial syrup, and it also has high alcohol content. The development of these tablets was implemented with the 3D-printing method using PEG 4000 as a plasticizer, which decreased Ritonavir’s T_g_. The 3D-printing method has the potential to modify the dose based on each child’s requirements. Moreover, tablets (solid state) have improved bioavailability and because of their small size, and children can swallow them more easily. The small size may negatively affect the dissolution of Lopinavir, however [103].

Ιn another study, the researcher’s aim was to find a polymer carrier that was suitable for an immediate-release delivery system in order for the drug to be absorbed from the gastrointestinal tract. In this case, the drug was Amiodarone, which has low aqueous solubility (between 0.2 and 0.5 mg/mL), which leads to problems in absorption. PEG (especially with an MW of 4000 g/mol) seemed to be an effective way to carry the model drug mainly due to the fact that it acts as a very good plasticizer and also has a hygroscopic semi-crystalline character. Hence, the use of PEG 4000 combined with Soluplus in a ratio of 3:1 allowed for prolonged disintegration. Furthermore, researchers used PEG 6000 as part of a copolymer [39].

Additionally, extended-release tablets of Diltiazem Hydrochloride (DTZ) were formulated by Tsuji et al. PEG 6000 played the role of a water-soluble filler that was incorporated either in HPMC or in PEO matrix tablets. Time-domain NMR was a helpful technique for the evaluation of the hydration behavior of the tablets that were immersed in water during the experiments. The results showed that PEG affected the behavior of HPMC and that around 63.1% of the active substance, DTZ, was released after 6 h, with DTZ being released steadily for at least another 8 h. In the case of PEO, the release profile was different, since a percentage of release of Diltiazem Hydrochloride of around 85.7% after 6 h was achieved [104].

#### 3.1.8. Fused Deposition Method

With the intention to develop tablets of Ibuprofen, researchers used PEG 6000, which is a semi-crystalline substance, and a polymeric matrix of ethyl cellulose to produce solid filaments of the drug molecule. Then it was used in 3D-printing technology (fused deposition), which was very useful to make the optimal formulation composition. The modification of the release can be beneficial for keeping the drug’s concentration in plasma constant, as well as for the reduction in side effects. In addition to this, due to this type of release, patients can easily comply with their treatment [105].

Furthermore, using fused deposition modeling for the development of intra-gastric controlled-release 3D-printed tablets of Pregabalin was a way to overcome the problems of short half-life of elimination and the need to be administrated two–three times daily. Researchers succeeded in increasing gastric retention, an important prerequisite for the absorption of Pregabalin, which took place mainly in the stomach and upper gastrointestinal tract (GIT). This increase resulted in a reduction in the doses that should be administered. In that case, PEG 400 was used as part of the filament that was the starting material of the fused deposition technology. The formulations made by this method have mechanical strength and zero friability. Moreover, the final formulation was floating and inhibited premature gastric emptying. The drug release fitted better to zero-order kinetics [106].

Moreover, pulsatile tablets (core–shell) of Verapamil hydrochloride were fabricated using the fused deposition method and achieved the release of the active substance within 4 and 8 h. The study reported the use of PEG with a molecular weight of 400 g/mol for shell fabrication with the role of a plasticizer that can adjust the thermoplasticity of the produced filaments. During that process, PEG remained stable. An important advantage of these formulations is that they have the potential to be used for personalized medicine through modification of the filament’s composition, geometric structures, and shell thickness [107].

#### 3.1.9. Tablets Formulated with 3D-Printing Technology

Three-dimensional-printing technology was very useful for the production of immediate-release tablets for the administration of Puerarin. In that case the model drug (Puerarin) formed eutectic mixtures with PEG with a molecular weight of 4000 g/mol, a polymeric carrier which is soluble and has a low melting point. In these mixtures Puerarin was dispersed in the crystalline state, and as a result, it improved the dissolution profile. Yet, the main advantage of this fabrication is that there is no need for solvents nor for a drying process [108].

Tomographic/volumetric 3D printing can print simultaneous tablets with two shapes, torus and cylinder. In a previous work, researchers used PEG-DA 575 or 700 (a photoreactive PEG derivative), which has the ability to crosslink, and PEG 300, which played the role of a non-reactive diluent that allowed for faster diffusion out of the solid matrix and into the liquid media. The results showed that it took between 6 and 14 h for the release of 90% of Paracetamol from torus tablets and between 12 and 15 h from cylinder tablets. By changing the ratio of photoreactive monomers and dilutants, it is feasible to change the release profile. However, despite its speed, this method managed to print only one item at a time and only symmetric items [109].

Another category of printing methods includes melting solidification printing processes, with which nanocrystal 3D-based tablets of a poorly soluble drug, Albendazole, were formulated successfully. Nanocrystals allow for high drug loading (concentration of up to 50%) and also their formulation consists of a scalable process. PEG 1500, which has a low melting point, played the role of a carrier. X-ray diffraction showed a relative crystallinity of nanocrystals with Albendazole equal to 86.13%, compared with a sample that had 100% crystallinity. However, there is a disadvantage to this method, which is the difficulty to orally administrate solid forms with nanocrystals [110].

In another study, Dronedarone hydrochloride, a drug for cardiac arrhythmias, was released in a prolonged way after 24 h by using PEG 20000 for the formulation of polymeric blends, where the drug was dispersed. An important advantage is that these polymeric blends are easily composed, and a quantity of 10% PEG is ideal for extrusion and printing. The application of the 3D-printing technology gives the potential of personalized therapy, and it is also beneficial for packaging precision [111].

A PEG derivative, polyethylene glycol diacrylate (PEG-DA) was used for the formulation of gastro-floating tablets containing, as active substance, Metoprolol tartrate. Through the extrusion 3D-printing method and with PEG-DA, the preparations made had tunable properties, as well as complex internal structures. PEG-DA is a photocurable polymer, with a long crosslinking time and low viscosity without toxicity, which can be easily modified. As for release from these tablets, the authors achieved sustained release for 12 h with a cumulative release rate equal to 90%. The final formulation contained different densities of the infill, and as a result, it has the potential of personalized therapy [112].

#### 3.1.10. Other Examples of Tablets

The objective of the research study by Liew et al. was to find a way to overcome the problem of patient noncompliance. PEG 6000 at various concentrations was tested as an additive in order to increase the hardness of the orally disintegrating tablets of Dapoxetine. However, corn starch and cocoa butter with the roles of additives for swelling and melting, respectively, were chosen as the optimal formulation. The final tablets had a reduced disintegration time of around 152 s and released 80% of the active substance after 30 min. Moreover, after exposure at 30 °C and 75% humidity, the Dapoxetine tablets remained stable after a period of time equal to 12 months [113].

PEG 4000 as a hydrophilic carrier improved the wettability and solubility of Ticagrelor (a poorly soluble drug), by converting it from crystalline to an amorphous state in the final formulation, the tablet form. This resulted in an increase in the bioabsorption of Ticagrelor, which was low because of the P-gp effect. The improvement in bioabsorption could also be beneficial for patient compliance, as well as for the reduction in side effects and therapy cost. As for the release it had quick onset and reached an acceptable percentage of release of up to 86.5% in 30 min [29].

Furthermore, the hydrophilic PEG 4000, acting as a meltable binder, endowed the final formulation of Lactose monohydrate tablets with self-lubricant properties. Moreover, direct compression was accomplished by the compression of excipients (based on fluid bed granulation) such as PEG or P188. As for the final tablets, consisting of 20% PEG 4000 and 20% POL188, they had acceptable strength, higher than 2 MPa, as well as good lubricant properties [114].

### 3.2. Solid Dosage Forms—Capsules

Capsules are also solid dosage forms where PEG could play a key role in their characteristics. In this section, special attention will be paid to the physicochemical characteristics of PEG that ameliorate the characteristics of capsules and the release profile of the encapsulated APIs.

#### 3.2.1. Capsules with Special Characteristics

The use of a soft-capsule-based self-microemulsifying delivery system was investigated as a way to increase solubility and also to orally administer Andrographolide. This active substance is poorly soluble, and there is a need of multiple dosage forms per dose. To achieve the above objectives, PEG 400 was used a cosurfactant to reduce rigidity and give more flexibility to the interfacial film of the surfactant. Moreover, PEG-400 was used as a solubilizer, and from the results in a gastric environment (pH: 1.2), the dissolution was completed in 30 min, compared with a maximum of 73% of dissolution in 2 h when using hard capsules filled with Andrographolide paniculata extract powder. Finally, the permeability through the intestinal membrane after 24 h of the formulated drug delivery system was equal to 827.87 ± 63.77 μg/cm^2^ [115].

In another study, PEG with the same molecular weight (PEG-400) had a different role in the formulation of capsules. More specifically it was the liquid medium in which Alpha Lipoic Acid (ALA) was solubilized. ALA has low solubility in water; in the presence of gastric acid, it is unstable, and it undergoes extensive first-pass metabolism (in the liver), which results in low bioavailability, up to 30%. Researchers intended to create a formulation of hard gelatin capsules that could be suitable for each patient. In the presence of PEG, the entire ALA content was released in 5 min, but with the addition of silicon dioxide, 85.37% was released by this time. However, with the addition of silicon dioxide, the leaking of the content was prevented [28].

Additionally, using biocompatible and biodegradable PEG-DA 258, researchers created a formulation of semi-permeable capsules consisting of biomolecules for the administration of Fibrinogen. PEG-DA also has an important advantage: it is hydrophilic, and as a result, it allows for the direct encapsulation of proteins and enzymes without adsorption onto the shell. However, there is a limitation on molecular weight, since proteins or enzymes may have an MW equal to or above 32.7 kDa [116].

In the study by Kunte et al., the researchers presented a way to substitute PEGylated drugs, mainly due to the hypersensitivity reactions that their use may cause and because of the process of sequestration that reduces therapeutic effectiveness. Hence, they manufactured a delivery system consisting of branched amphiphilic peptide capsules that were associated with mRNA. The main advantage was that it improved the delivery of the drug through the cells without causing adverse effects such as cytotoxicity, oxidative stress, or changes in metabolism. Moreover, the capsules did not lead to chronic inflammation and were not disrupted by chaotropes or high temperature [117].

PEG with a high MW of 20,000 g/mol was part of the shell of microcapsules that had two parts, the core and the shell. It played the role of a carrier blended with PLA in a ratio of PLA/PEG equal to 6:4, and both were clinically safe without cytotoxicity. Τhe manufactured formulation achieved a (cumulative) release percent of 66% of the active substance, Nifedipine [27].

The work by Haimhoffer et al. constitutes another example of using PEG for the fabrication of capsules, in this case hard gelatin capsules including, as active substance, Curcumin. PEG was part of a polymer system that functioned as a matrix for oral administration. This matrix was a crosslinked matrix between the water-soluble polyethylene and β-cyclodextrin polymer, and it could be used for drugs, which are poorly soluble. As for Curcumin, the release was complete after 1 h, and the formulation led to a 12-fold increase in permeability. Moreover, the bioavailability was improved, and as a result, this could reduce the number of applied doses needed. Finally, PEG seemed to equip the matrix with mucoadhesion and complexation abilities [118].

The research study by Li et al. aimed at the fabrication of nano-capsules with responsiveness to Sulfur Dioxide (SO_2_), since the release of payloads could be accelerated by the presence of the oxide. A total of 38% of the drug SN-38, which has anti-tumor activity, was released after 12 h using PEG as a part of the PEG45-b-PVPOP14 copolymer. The above copolymer acted as a vehicle with sulfite responsiveness that can be used for the encapsulation of hydrophilic as well as hydrophobic matrices [119].

For the administration of a hydrophilic drug, Doxycycline hydrochloride and a hydrophobic substance, Meloxicam, PEG 1000 played the role of an additive that adjusted the melting point of the drugs and affected the release profile. Hence, researchers fabricated a system consisted of lyotropic liquid crystalline hard capsules to meet the demands of the formulation of drugs with different solubility and hydrophilicity. The structure of this system enabled a transformation into a liquid, hexagonal phase that allowed for a profile of 24 h sustained release. This change can be triggered by two factors: either the presence of water or the increase in the temperature to match that of the body. Nevertheless, at room temperature they were in a semi-solid state that allowed for a reduction in storage instability [40].

#### 3.2.2. Targeted Administration

Targeted delivery of drugs was accomplished through the administration of PEG capsules that were fabricated using emulsion droplet templating. The purpose of the above was to decrease the non-specific interactions of Doxorubicin as well as overcoming the biological barriers that exist. The final formulation consisted of soft capsules with a size of several micrometers. PEG methyl ethyl acrylate 480, PEG dimethyl acrylate, and also 8-arm PEG acrylate were used for the modification of the nanocarriers. From the results it was evident that this method was efficient in the delivery of the drug, since it reduced cell association and endowed the final formulation with biocompatibility [120].

In another study in which the researchers intended to deliver the model drug Fluorescein sodium salt to the colon, they used PEG with a molecular weight of 10,000 g/mol. In detail, they produced Polycaprolactone/Polyethylene Glycol capsules, since Polycaprolactone is a gastro-resistant polymer and PEG formed a permeable structure that modified the release from the capsules. A *w*/*w* ratio of PCL/PEG equal to 70/30 allowed for the release of the drug within around 60 h, which is an acceptable time for drug delivery to the colon [121].

#### 3.2.3. Coated Enteric Capsules

PEG monoethylether with an MW of 2000 g/mol was effectively used as a component of the copolymer PLGA-Hyd-PEG (nanoparticles) for the oral administration of coated enteric capsules of Insulin. In detail, these tablets were stable in the gastrointestinal tract and achieved a glycose reduction of up to 35% for a period of time of up to 10 h. PEG took part in the fabrication of a core that was sensitive to pH changes, and also, it converted the surface of the nanoparticles from hydrophilic to hydrophobic. Nevertheless, the experiments were performed in rats [122].

**Figure 4:**
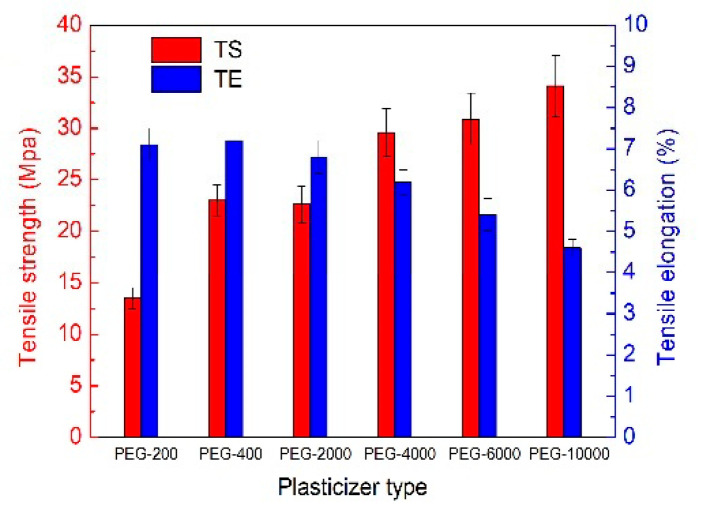
Effect of molecular weight of PEG on mechanical properties of films [41].

PEG-400, acting as a plasticizer, was investigated for its potential use in medicine or food supplements as part of enteric polymeric films. The purpose of that was to enhance the barrier and mechanical properties of the films, and PEG-400 showed to improve both plasticity and the water vapor barrier property. Compared with other plasticizers, such as glycerol, fructose, sorbitol, and beeswax, PEG-400 showed greater hydrogen bonding interactions with polysaccharides in the film, and it had lower water vapor permeability. Finally, the researchers studied how molecular weight affected the mechanical properties of the plant-based enteric capsules. The increase in the molecular weight of PEG led to higher tensile strength but lower tensile elongation, as is evident from Figure 4 [41].

In addition, PEG of 4000 g/mol was used in industry for the fabrication of hard uncoated enteric capsules of Pantoprazole that had the property to remain stable in the stomach, and after reaching the intestinal epithelium, they released the drug in a time of 10 min. In this study Polyethylene Glycol acted as a filler, and its use resulted in a decrease in the contained moisture and porosity. This is the reason for the stability of Pantoprazole in the stomach for 120 min [123].

Another study was implemented with the purpose of the fabrication of enteric capsules for the targeted release of Paracetamol, a soluble drug which is commonly used. To meet the demands of this project, which was to achieve Paracetamol release close to the intestinal epithelium, researchers used self-unfolding foils including a solid-state formulation. PEG 6000 was a plasticizer in this solid state, and the trial to use it as a coating material indicated that it increased both the flexibility and the integrity of the coatings. Moreover, Polyethylene Glycol increased the release rate of the drug. At pH 2.5 the percent of Paracetamol that was released was approximately 30% in 2 h and reached 75% in 13 h. Thus, the active substance was protected in the gastric environment, and at pH 7.5, the release of Paracetamol was complete within a period of 80 min [42].

The aim of the investigation by Shaqour et al. was to modify the release of Ketoprofen, which has low solubility and bioavailability, and for this, they produced capsules with PEG 400 as part of a HPMC: PEG filament, to make them sensitive to pH. This is very important, because the main problem of Ketoprofen is that it has side effects in the upper GI tract. By using PEG-400 as a plasticizer (which reduces the T_g_ of HPMC) the formulation managed to release the drug mainly in the small intestine and, as a result, reduced the possibility of gastric and duodenal ulceration. The release of the drug was enteric with a sustained kinetic profile. Finally, another advantage of the 3D-printing technique is that it is based on each patient’s requirements [124].

#### 3.2.4. Solid Dispersions

Low solubility of Albendazole, which leads to poor absorption after oral administration, can be modified by using PEG 4000 or PEG 6000 as solubilizers (PEG 6000 also as a carrier). However, in the final formulation of the solid dispersion, they used as solubilizers a combination of PEG 6000 and P888 in a ratio of 1:2 that led to a drug release of a maximum of approximately 95%. Moreover, the researchers intended to increase the exposure time of the Albendazole metabolites and mainly the time of the more active form, Albendazole Sulfoxide (ABZ-SO). From the results it is evident that they achieved that, since the relative bioavailability, compared with the commercially available counterpart, was, for the ABZ solid dispersion, 2.25-fold higher and, for the Albendazole Sulfoxide metabolite, 1.89-fold higher [43].

### 3.3. Nanocarriers

In this part of the manuscript, the usage of PEG in various types of nanocarriers will be analyzed in depth.

#### 3.3.1. Liposomes

PEG is used in drug delivery due to its antigenic effect against certain viruses, as well as its biocompatibility. In a previous study, PEG with a molecular weight of 2000, stable linkage with DSPE lipids, and a size of approximately 94 nm, when attached to the surface of liposomes, increased their stability inside the body and improved their circulation time; this led to sufficient levels of liposomes which allowed them to reach the target, making the delivery system more efficient. Despite its increased biodistribution, however, it is vital to ensure the correct dosage of PEGylated liposomes during the administration process because there are cases of high dosage leading to forms of anergy, making the immune system less tolerant to unknown encounters [125].

In order to achieve lower toxicity levels of liposomes and make them more unilamellar, researchers developed mPEG conjugated with DSPE and made the conjugate adhere to the surface of liposomes, providing a stable ether linkage that improved their circulation time in the kidneys. Having a significant weight distribution and a neutral charge, the PEGylated liposomes were proven unharmful towards the body’s cells and led to large drug encapsulation value. Using Doxil as the pharmaceutical ingredient, the nanobodies possess great anti-tumor properties and increased in vivo stability. Without the PEGylated liposomes, Doxil itself can cause hypersensitivity and decreased tumor accumulation, highlighting the importance of the nanobodies throughout its delivery process [126].

Another study showed that PEGylation (adjoined with DSPE to form DSPE-PEG5000 molecules) of DPPC and cholesterol liposomes increases the stability of the delivery nanosystem, resulting in easier flow inside the body, as well as increasing retention in the lungs, making the active pharmaceutical ingredients (Metformin and Nintedanib) more effective against lung injuries like Pulmonary Fibrosis while providing protection against bacteria and longer-lasting survival in the body. Their uniform size and negative zeta potential, as well as their ability to achieve a narrow weight distribution, make them potential drug delivery systems with enhanced antifibrotic properties [127].

Furthermore, while assessing the value of PEG as a highly biocompatible molecule, researchers recognized its ability to be easily accumulated on the surface of liposomes for high Nafcillin loading in healthcare settings. PEG improved the antibacterial effects of the drug-loaded liposomes, enhanced their targeting ability against the infection viruses, and increased their stability in vivo. Possessing a loading value of over 90% and a significant drug concentration inside the body, the drug carriers represent a great asset against infections, with PEG providing liposomes with low polydispersity (PDI of 0.216), negative zeta potential (−38 mV), and high permeability [44].

Focusing on the effectiveness of DSPE-TK-PEGylated DMF liposomes as drug delivery systems carrying Dimethyl Fumarate as an antifibrotic agent and utilizing the liposomes’ biocompatibility and clinically proven safety, as well as PEG’s hydrophilicity and low toxicity values, which do not trigger the organism’s immune system, research showed that PEGylation aids in prolonging retention time in the lungs, thus achieving higher effectiveness in combating Pulmonary Fibrosis. The spherical shape and low negative zeta potential gave the nanosystem high encapsulation efficiency and drug loading values reaching up to 90% and 8.5%, respectively, but during drug release, the delivery faced problems with a lack of homogeneity due to damage to the hydrophilic layer, causing debris of nanoparticles to scatter and leading to lower control of drug release [4].

To further dive into the topic of PEG surface-functionalized liposomes, an investigation used etheric PEG, which is characterized by great stability, and liposomes derived via lipid peroxidation to capture Ferrocene in liposome hydrophilic core as an iron source for cancer therapy against Tumor Ferroptosis. With a molecular weight of 2000 and an IC50 value of 142 μM, PEG successfully provided the liposomes with a narrow size distribution, proven by the size polydispersity index, calculated as 0.25, while also making them suitable for intravenous administration by creating stable fluids that mimic the body fluid environment. The only problems faced were low loading efficiency, with the mass of the drug being only 8.8% of the total mass of the delivery system, and the inability of the liposomes to kill various cancer cells [45].

Amoxicillin is an antibacterial drug which is clinically proven for its effectiveness against several infections but which has the disadvantage of being unable to counteract all the resistance mechanisms that were created by the abuse of antibiotics. For this purpose, researchers studied the added value of PEG as a surface-attached molecule on liposomes while surviving the attacks of the immune system of the human body. PEG is considered a stable polymer with high water solubility and moisture retention whose binding onto the liposomes creates a supramolecular network that minimizes the movement of water, thus decreasing swelling. With a size of 210 nm and a polydispersity index of 0.26, the PEGylated liposomes were proven mechanically robust, indicating that a great application to counteract resistance against antibiotics has been created [46].

In an article by Minamisakamoto et al., liposomes with a spherical shape and pH suitable for drug loading were coated with PEG2000-DSPE for the transfer of Doxorubicin inside the body. Also, in this case, PEG contributed as a stable polymer with high permeability that added to the efficiency of the liposomes as drug delivery systems by making them flow easily through the body. The calculated encapsulation efficiency value of 98% leads to the assumption that the physicochemical properties of the PEGylated liposomes, like the low zeta potential (−0.06 mV), the low polydispersity index (0.19), and the nanometer size (136 nm), played a vital role in creating a steady environment for the release of Doxorubicin, thus supporting an increased anti-tumor effect. Despite the sufficient loading efficiency, the results showed that lower concentrations of the API increased the effect, implying that the strong retention of the API can deteriorate the controlled release [47].

PEG’s hydrophilic properties show great potential in the delivery of Doxorubicin due to its binding onto the surface of liposomes and its amplification of their physicochemical properties for a greater anticancer effect. In a study by Maghsoudi et al., the researchers focused on the clinical study of these delivery nanosystems. Taking advantage of the thermosensitivity and the magnetic nature of liposomes and with the significant impact of PEG conjugated with Fe_3_O_4_ on maintaining stability during the transfer and increasing the circulation time within the blood, the results showed that the nanosystem had a negative zeta potential value of −61.7 mV, a size of 133.2 nm, and a polydispersity index of 0.296. These results indicate that PEGylation contributed to the mass of the loaded drug being over 75% of the initial mass, thus resulting in successful Doxorubicin encapsulation and delivery. The only drawbacks were that the transfer was slow and that the nanocarrier was not responsive without the correct composition of materials [48].

Another article, written by Tang et al., examined the encapsulation of Doxorubicin in the aqueous core of liposomes with PEGylation, which provided the liposomes with enhanced physicochemical properties. PEG was attached to the surface of the liposomes, creating a biocompatible delivery system with low toxicity. The hydrophilic nature of PEG contributed to prolonged circulation in vivo along with high loading capacity, with the size of the nanocarriers being over 120 nm, and a wide weight distribution, with a polydispersity index of over 0.17. PEGylation offers a significant tool in the field of drug development and encapsulation, although it faces certain obstacles during quality control due to weaknesses in SPE analysis, including errors caused by overconsumption of the organic ingredients or leakage of the carrier drugs [49].

#### 3.3.2. Micelles

Regarding the study of micelles, entrapping Paclitaxel inside PEGylated micelles and injecting the latter into the circulation was proven useful in order to induce a pharmaceutical effect during cancer treatment. Paclitaxel was loaded in the aqueous core of micelles which were formed by PEG-b-PLA copolymers for improving their capability to dissolve the hydrophobic drug and assisting in a more efficient delivery. The nanosystem, with a size of 40–50 nm and a size polydispersity index of 0.18, showed prolonged circulation in the blood while also maximizing the quantity of Paclitaxel, with the mass of the loaded drug being over 90% of that initially added [50].

Combining PEG’s hydrophilicity, biocompatibility, and biodegradability in PLGA1200-PEG1450-PLGA1200 triblock copolymers with polyurethane micelles, which provided reactive sites, was enough to set the goal of enhancing the targeted delivery of Gliclazide. The combination resulted in a zeta potential value of −4.5 mV and a size distribution of 0.258 (PDI) leading to the mass of Gliclazide being over 12% of the total delivery system. Ensuring encapsulation efficiency over 60% led to successful targeting and release regulation [51].

In the research study by Feng et al., the authors focused on the effect of DSPE-PEG2000 on treating tumors by using the anti-inflammatory properties of Amentoflavone trapped in micelles. PEG’s added value included enhanced stability and increased blood circulation time while also promoting efficient accumulation at the tumor site, all proven by the high encapsulation efficiency value of 98.80% and the zeta potential value of −27.67 mV. PEG also successfully created a reduced toxicity environment with IC_50_ values as low as 4.74 μg/mL and 9.43 μg/mL in the stomach and lungs, respectively, thus proving its significance in the drug delivery process [52].

Considering the investigation of micelles, researchers took advantage of their spherical shape in the drug delivery of Methotrexate by capturing it inside their hydrophobic core, increasing bioavailability. Despite the great physicochemical properties of these micelles, PEGylation was also implemented to provide them with biodegradability and biocompatibility, as well as great water solubility, in order to create an amphiphilic environment for cancer treatment, forming PEG-b-PCL micelles. With a hydrodynamic size of 122 nm and polydispersity index of 0.42, PEG successfully assisted in achieving a more stable delivery nanosystem with increased invisibility to the immune system, thus reaching the target more effortlessly [53].

Moreover, Curcumin’s anti-inflammatory and anti-tumor effects reduce damage inside the liver, while trapping a drug in TPGS micelles helps create delivery nanosystems for boosted administration to the human body. These systems were improved by attaching Gal-PEG-PLA copolymers to the surface due to its hydrophilic nature and ability to solubilize drugs for greater entrapment, proven by the high loading efficiency of 20.3%. The calculated negative zeta potential and relatively low size provided the nanocarriers with efficient blood circulation, making them suitable for oral absorption while also successfully entrapping more than 85% of the API in the micelles [54].

In the article written by Du et al., which similarly focuses on PEGylated micelles, the research study was based on mPEG-MAL micelles and drug delivery. Keratin micelles possess the ability to capture hydrophobic drugs like Doxorubicin into their hydrophobic core by increasing their water solubility. PEG has a hydrophilic structure with reduced cytotoxicity and a narrow molecular mass distribution. The research study showed that PEG prolonged the blood circulation time while limiting immunogenicity, as shown by the low IC_50_ value of 14.13 μg/mL. The polarity of the PEGylated micelles with a zeta potential value of −25.2 mV and a polydispersity index of 0.4 caused a high total loading of the API, thus proving their effectiveness as a drug delivery system [55].

Within the scope of drug delivery, Doxorubicin can be entrapped inside spherical micelles (consisting of palmitoyl-pMPC polymers) due to their amphiphilic nature and their ability to carry the API inside the human body. For enhanced delivery, PEG (conjugated with Polylactic Acid and Polycaprolactone) was studied for its significance in prolonging the circulation time of the drug-loaded micelles inside the human body and providing them with low-cytotoxicity properties. The obtained size of the micelles was 16.7 nm, which makes it easy for them to navigate inside the human body in order to achieve a more targeted drug delivery, although the delivery caused certain hypersensitivity reactions leading to anaphylactic shocks [56].

Another macromolecule investigated is mPEG-PDLLA, capable of bonding with micelles in order to assist during targeted drug transfer and release of Epirubicin. It was scientifically proven that attaching PEG to the surface of micelles composed a shield against the immune system, improving the ability of the nanosystem to circulate inside the body while also providing great stability. Achieving an encapsulation efficiency of over 70% and a negative zeta potential, PEGylation led to the accumulation of a significant quantity of the drug at the targeted site, also proven by the IC_50_ value of 7.03 μg/mL, making the PEGylated micelles valuable in the field of drug delivery [57].

Although micelles have been clinically researched as drug carriers inside the human body due to their ability to load Arenobufagin because of their hydrophobic tails and hydrophilic surface, binding PEG-PLA copolymers and micelles led to improved physicochemical properties, with the hydrophilic PEG providing enhanced accumulation of the drug at the targeted site by avoiding its clearance from the circulation by the immune system. This was proven by encapsulation efficiency values as high as 82.5% and loading efficiency of 7.5%. PEGylated micelles also had a size of 97 nm and a polydispersity index of 0.104, rendering them biocompatible while also gaining the high water solubility that PEG provides [58].

Lastly, an article by Singam et al. focused on the use of spherical micelles loaded with Doxorubicin and how PEGylation of the micelles is beneficial. The API is trapped inside the aqueous core of the micelles, while EC-PEG copolymers work as stabilizing agents by getting attached to the surface. Due to the low immunogenicity of PEG and its biocompatible properties, the delivery system attains improved shielding from the immune system during its circulation in the blood, making the transfer more efficient while also resulting in high permeability within tissues. The prolonged blood circulation, along with the stability of the nanocarriers, highlights the importance of PEG during drug delivery while also raising interest in micelle-based delivery systems [59].

#### 3.3.3. Polymersomes

Polymersomes can encapsulate hydrophobic drugs like Hypericin into their aqueous core and get coated with PEG-b-PCL for great biocompatibility and biodegradability. PEG is attached to the hydrophilic surface of polymersomes in order to improve their pharmacokinetic and pharmacodynamic properties. As far as the body’s reaction is concerned, PEG is proven to prolong the circulation time inside the blood vessels, thus avoiding clearance by the immune system. As far as the nature of the nanocarriers is concerned, PEG creates a stable formation with a narrow size distribution (size of 183 nm and polydispersity index of 0.140) by creating multiple thick membranes for efficient drug transfer. The calculated encapsulation efficiency was 98.7%, and the loading efficiency was 8.7%, proving the efficiency of the delivery nanosystem, although experiments have shown that there have been disruptions to the morphology of the polymersomes during sizing procedures, which had led to unwanted release before the target tissue was reached [60].

Additionally, polymersomes have a core–shell-like structure which enables them to entrap APIs like Doxorubicin and provide a shielding factor during transfer. To enhance their physicochemical properties, PEG-PCL copolymers provide the added value of biocompatibility, while fabricating a versatile nanosystem with a large size and a wide weight distribution. The high absolute value of the zeta potential (−30 mV) indicates stability, making the way for sustained transfer and release of the API [61].

In an article written by Khodaverdi et al., the authors focused on the transfer of Docetaxel in the human body against cancer during chemotherapy, where polymersomes acted as the transferring vehicle due to their loading capacity and amphiphilic environment. PEGylation was implemented to further enhance the process of drug delivery through PEG-PCL and PEG-PLA copolymers by assembling superficially into polymersomes and improving their physicochemical properties. The added value of the formulation was noted as improved biocompatibility and drug solubility while also providing stability and reduced side effects like toxicity (IC_50_ 11.79 μg/mL) and clearance by the immune system. The zeta potential value of −12.7 mV and the polydispersity index of 0.262 led to high permeability, while the high encapsulation efficiency proved the effectiveness of the delivery nanosystem [62].

Another study covered Simvastatin and BDNF as anti-inflammatory agents, which face problems during administration, like reduced expression, and the researchers attempted to prevent their side effects. In order to preserve the therapeutic properties of these agents, the administration proceeded via encapsulation inside the aqueous core of PEG-PdLLA polymersomes. Polymersomes are excellent transfer vehicles due to their amphiphilic environment and spherical shape, which assist in drug entrapment, while PEG provides biocompatibility, versatility, and stability when it is chemically bound onto the polymersomes. PEGylation has been proven to secure the functionality of the drugs while also contributing to amplifying its physicochemical stability. The size of 265 nm, the polydispersity index of 0.2, and the zeta potential value of −20 mV provide proof of efficiency and support the safe delivery of the drugs [63].

PEGylation is implemented in drug delivery to provide polymersomes with functional groups that help the nanosystem adapt inside the circulation and avoid clearance by the immune system, thus prolonging the circulation time. PEG forms copolymers with PLA (of the (PEG)_3_-PLA type), which, along with their high density and large length, create a shield around the polymersomes, rendering them stable and inhibiting the uptake of colloidal nanoparticles during the delivery of APIs [64].

Despite polymersomes having a versatile morphology and possessing the ability to load drugs like Doxorubicin inside their hydrophobic core, in order to improve their physicochemical properties, PLGA-PEG-PLGA attachment onto their surface has been proven quite successful due to PEG’s longer-lasting stability and biocompatibility. Except for the downside of the low permeability of hydrophilic drugs, PEGylation manages to increase the circulation time inside the blood vessels while also preventing the accumulation of proteins. Achieving a size of 174 nm and a zeta potential value of −20.44 mV, PEGylated polymersomes showed high potential during drug delivery, with clinically calculated mass accumulated in the body being over 50% [65].

During the drug encapsulation of Carboxy fluorescein, PEG-PLA and PEG-PCL copolymers enhance the pharmacodynamics and pharmacokinetics of polymersomes that face problems like low stability and biodistribution. PEGylation is implemented to provide biocompatibility and lower the polydispersity index to under 0.2 due to its low molar mass distribution. Also, PEG is used as a surface shield against the immune system for longer circulation and for supporting the nanosystem throughout the delivery process. Despite the significant lab results, PEGylated polymersomes have not been successfully administrated to a patient yet [66].

Although an API like Fisetin is characterized by low solubility in water as well as low bioavailability, scientists developed PEG-(PCL)_2_-based polymersomes that transfer Fisetin into the human body and the target site while providing stable delivery. In particular, PEGylation of the nanocarriers improved the solubility of the API by creating a hydrophilic environment, as well as providing reduced toxicity and advanced circulation in the bloodstream due to the small size of the polymersomes (47 nm), making it possible to pass through the vessels. The calculated loading efficiency of 7.1% proves the potential of the PEGylated polymersomes as delivery agents of Fisetin that can assist in improving the physicochemical properties of the system [67].

On another occasion, breast cancer was treated with Doxorubicin entrapped in polymersomes with PEG-PLA attached on the surface, acquiring increased safety as an encapsulation nanosystem. Polymersomes provide an amphiphilic structure that can capture the API into their aqueous core, while PEG provides biocompatibility and hydrophilicity. PEGylation improves the flow of the nanosystem in the bloodstream by creating a shielding mechanism that ensures reduced recognition by the immune system, as proven by the low zeta potential (−0.27 mV) and the low polydispersity index (0.15). Achieving high drug encapsulation (68%), polymersomes managed to successfully assist in the delivery process against breast cancer [68].

Overall, polymersomes possess high amphiphilicity, which aids in Doxorubicin encapsulation in order to boost its pharmacological effect. Surface-bound PEG-PLA copolymers have also been developed as shielding agents for drug transfer into the human body. The high hydrophilicity and permeability provided by the size distribution of PEG (size of 79.71 nm and polydispersity index of 0.15) leads to significant drug uptake (95%) and low leakage, as well as easy flow through the blood vessels due to the acquired invisibility to the immune system [69].

#### 3.3.4. Inorganic Nanoparticles

PEG is used in the drug delivery of Doxorubicin for its hydrophilicity and high water solubility, which make it attachable to magnetic inorganic nanoparticles (e.g., NiFe_2_O_4_, CoFe_2_O_4_, and Fe_3_O_4_), providing the carrier system with invisibility to the immune system, as well as biocompatibility and reduced antigenicity. PEGylation is proven to reduce the toxicity levels and enhance the magnetic properties due to the ionization provided by the aqueous environment [70].

Another example is given by graphene oxide–silver nanoparticles, which were developed due to their ability to provoke magnetic interactions with Curcumin, inducing its pharmacological effect and transferring it to the desired tissue of the body. PEGylation acted as a stabilizing strategy that prevented the oxidation of NPs by coating the surface while also providing moisture retention due to its great water solubility and hydrophilic nature. The size of this particular drug delivery system was approximately 10 nm, and the system was characterized by great stability and did not trigger decomposition by the immune system or cause harm to the organism [71].

Inorganic nanoparticles have thriving potential for the encapsulation and delivery of Doxorubicin due to their abilities to bond electromagnetically to it. PEGylation is used to enhance the pharmacokinetics and pharmacodynamics of nanoparticles through bonding with PEG-PLA copolymers by creating a hydrophilic environment for improved stability. The density of this nanosystem (polydispersity index of 0.09 and zeta potential of −5.4 mV) leads to prolonged circulation within the blood vessels and the ability to reach the targeted site more efficiently, thus reducing the oxidation of the NPs, as well as assisting in drug transfer. PEGylation also provides decreased immunogenicity in order to avoid rapid clearance by the immune system, although it was proven quite challenging to control the morphology of the nanosystem for sustained release [72].

In addition to the above, scientists investigated the potential of inorganic Zn nanocarriers for entrapment of Shikonin and its transfer to the target tissue. By binding with PEG, the nanosystem acquired low toxicity, and scientists aimed at enhanced anti-inflammatory effects by creating a biocompatible and safe material. PEGylation gave the nanosystem improved water solubility and prolonged cell viability. The nanosystem was small in size (30 nm), rendering it effective in terms of passing through the body for quick delivery [73].

It is known that PEGylation can be achieved by binding PEG onto the surface atoms of inorganic magnetite nanoparticles conjugated with polymyxin-B and can act as a shielding agent that assists in navigating inside the body without being cleared by the immune system. Inorganic particles obtain the advantage of enhancing the antibacterial properties of drugs by capturing them magnetically, and PEGylation provides reduced toxicity and increased stability, as well as reducing the side effects [74].

By the same token, using ferrous inorganic nanoparticles was proven effective in drug delivery on account of the magnetic abilities and the electrical charge obtained, which allow for their easy penetration into tissues and high permeability (zeta potential of ca. −0.63 mV). By chemically combining PEG with the inorganic nanoparticles, the delivery nanosystem acquired increased stability, reduced toxicity, and prolonged circulation inside the body. The size of 650 nm and the polydispersity index of 0.163 were proven useful assets for effective targeted drug release without nanosystem disintegration and with improved invisibility to the body’s immune system [75].

While studying the advances in drug delivery, research showed that Doxorubicin’s anticancer abilities and high affinity binding, along with the use of gold nanoparticles, constitute a potential versatile delivery system with low toxicity for greater anticancer effect. In this case, PEGylation was also implemented for enhanced solubility and stability, as well as invisibility while travelling inside the body, thus allowing for permeation of the nanocarrier without it getting annihilated by the immune system. The small size of 21.50 nm provided the nanocarriers with enhanced permeability and versatility, leading to more controlled drug release [76].

Alongside significant biocompatibility and cytotoxicity, PEG also contributes physicochemical properties while being attached onto inorganic Au nanoparticles. This nanosystem is used in drug delivery because of its small size (90 nm), which allows it to be mobile inside the blood vessels, thus reaching the target tissue effortlessly while also being stable for increased accumulation. Despite pH sensitivity, PEG acts as a shielding agent due to its large surface, which ameliorates flow during delivery, as also proven by the low zeta potential value of 0.6 mV [77].

Another example includes silica nanoparticles, which acquire electromagnetic properties that enhance the inherent properties of the nanosystem while encapsulating Doxorubicin for targeted drug release. mPEG is again a coating component that provides high absorption and helps the nanosystem pass with ease through body tissues. The negative zeta potential value and small size allow for enhanced permeability and reduced toxicity provided by the non-immunogenicity of PEG while also attaining biocompatibility, although there have been cases of PEGylated drug-loaded inorganic nanoparticles that have caused allergic reactions [78].

Lastly, in order to create a sustainable nanosystem for the delivery of Crocetin to living tissues, the drug was entrapped inside magnetite nanoparticles using magnetic forces, and the nanoparticles were coated with PEG for enhanced biocompatibility and stability. PEGylation led to a significant encapsulation efficiency of 90%, thus assisting in the transfer through the body and avoiding clearance by the immune system. The IC_50_ value calculated as 46.2 μg/mL is a sign of limited immunogenicity; the nanosystem also presented with good water dispersibility caused by the hydrophilicity of the PEG coating [79].

#### 3.3.5. Niosomes

In an article written by Hosokawa et al., it was disclosed that PEGylation of Span niosomes provided them with a hydrophilic outer shield/corona, which allowed the niosomes to navigate through the blood vessels more easily without triggering the immune system while also preventing tumor enlargement. Despite the slow release of the drug at the target site, the size of 273 nm and the zeta potential of −43.2 mV ensured physicochemical stability and promoted high drug encapsulation (85.8%), proving the significant potential of PEG in the field of drug delivery [80].

**Figure 5:**
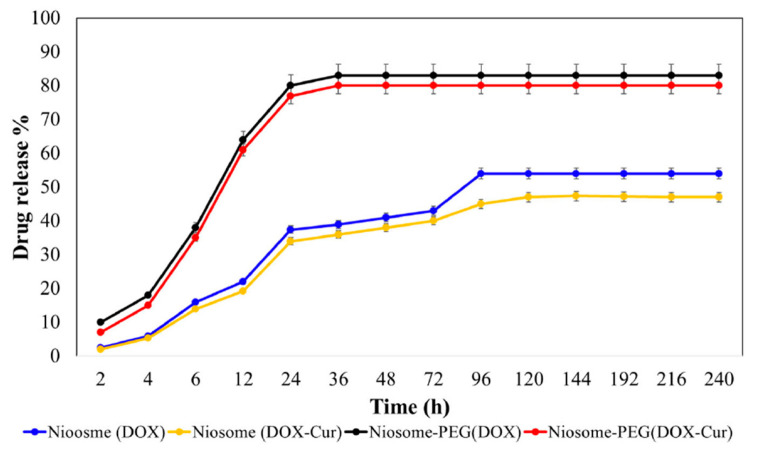
PEG contribution to drug release rate of niosome-based nanosystems [81].

PEG_2000_ has contributed to research on controlled drug release (Figure 5). It was proven to prevent the premature release of Curcumin captured in Span 60 and Tween 60 niosomes by providing stability, as proven by the low zeta potential and size polydispersity index. The total mass of the loaded drug was 96.5% over the non-loaded nanosystem, and the IC_50_ was 20.7 μg/mL, indicating that the addition of PEG on the surface of the delivery nanosystem improved its physicochemical and pharmacological stability [81].

In drug delivery, PEGylation was employed for enhanced pharmacological effect, as it provides stability and higher permeability while also avoiding clearance by the body’s immune system. The low toxicity, the biocompatibility, and the non-immunogenicity of PEG contributed to creating a stable delivery nanosystem that included niosomes, made of alkyl and dialkyl polyglycerols, with improved physicochemical properties (zeta potential of −32.7 mV, size of 241 nm, and polydispersity index of 0.20) and solubility. The added value was proven by the high encapsulation efficiency of 91.7% [82].

Regarding acetaminophen, studies have shown that it presents high toxicity rates through simple administration and that its toxicity can be balanced through the use of PEGylated niosomes. Span 60 niosomes entrapped the API inside their aqueous core while PEG acted as a shielding agent for longer-lasting stability. It was proven that PEG managed to improve the in vivo properties of the delivery nanosystem by avoiding the interactions with the pre-existing biological molecules inside the body tissues and obtaining a zeta potential as low as −23.9 mV for higher permeability. The niosomes showed high biocompatibility and biodegradability, with a size of 242.3 nm, thus manifesting potential as nanocarriers of APIs [83].

Generally, incorporating drugs into the aqueous cores of nanocarriers like Span 60 and Tween 60 niosomes has been significantly studied by scientists for controlled-drug-release procedures. PEGylation of the niosomes is a way of increasing the solubility and permeability of the nanosystem in vivo while also promoting oral administration with low toxicity rates and prolonged circulation time. Creating physicochemically stable structures with an average size of 156 nm has shown potential for drug delivery, with PEG_600_ providing enhanced cytotoxicity against cancer cells, apart from being compatible with the safety protocols for administration [84].

Nisha et al. focused on the clinical performance of PEGylated niosomes containing b-sitosterol and discovered that using PEG_2000_ to coat Span 60 and Span 20 niosomes provided them with stability and longer-lasting survival in the body while also improving the cytotoxicity of the system against cancer cells. Obtaining significant physicochemical properties like a low polydispersity index value of 0.078 and a zeta potential value of −19.54 mV, PEGylation led to efficient drug loading, reaching up to 16.72%, as well as a high encapsulation efficiency of 78.04% [85].

Additionally, PEG_2000_ was attached onto the surface of Span 20 niosomes with increased stability and helped the nanosystem flow easily inside the body. Proven to assist in leakage prevention while also providing protection against bacteria and longer-lasting survival in the body (IC_50_ 22.5 μM), the nanosystem showed a size of 248.5 nm and a polydispersity index of 0.25, which ensured high encapsulation of the API (95%) and achieved the successful loading of Carboplatin, with a calculated percentage of 21% [86].

For the transfer of Tamoxifen, PEG_6000_ provided the added value of stability, inducing invisibility to the body’s immune system and fabricating a versatile niosomal nanosystem with a determined size of 582.70 nm and a zeta potential of −38.70 mV. The high encapsulation efficiency of 88.73% indicates the efficiency of the delivery and the minimization of unwanted side effects [87].

Adding to the abovementioned studies, it was shown that PEG_2000_ is a stable polymer with high permeability that contributes to drug delivery by making Span 60 and Tween 60 niosomes flow easily through the body and simplifying the transfer of entrapped Gingerol without being eliminated rapidly by the immune system. The encapsulation efficiency value of 73.29% leads to the assumption that the physicochemical properties of the PEGylated niosomes, like the zeta potential (−12.3 mV) and the size (121.65 nm), played a vital role in creating a steady environment for drug entrapment and release (IC_50_ of 88 μg/mL), thus showing an increased anticancer effect [88].

Lastly, Tween 60 niosomes coated with DSPE-PEG were developed due to their increased safety as encapsulation mechanisms for greater inhibition. These niosomes were self-assembled nanoformulations with a spherical morphology that can entrap Curcumin into their core, while PEG provided biocompatibility and hydrophilicity. PEGylation reduced the particle size (117.5 nm) and decreased the release rate, thus allowing the system to transfer the drug more effectively, with the process also being enhanced by the low zeta potential (−15.1 mV), leading to high drug encapsulation (72%) [89].

#### 3.3.6. Nanoparticles

With a molecular weight of 5000, biocompatible PEG successfully provided Horseradish Peroxidase-loaded liposomes with a linking site for ligands in order to improve cell targeting inside the brain while also rendering them invisible to the immune system, thus prolonging circulation inside the vessels. The low toxicity levels were indicated by the low IC_50_ value of 7.70 μg/mL, suggesting the importance of the PEG outer corona in controlled drug delivery [90].

Chemically combining PEGylated nanoparticles conjugated with Folic Acid and Chitosan for enhanced targeted drug release led to higher stability, enhanced accumulation at the targeted site, and prolonged circulation inside the body. The size of 113.55 nm and the polydispersity index of 0.28 were proven useful characteristics for effective targeted drug release, with PEG contributing to improved physicochemical properties by raising the solubility level and providing hydrophilicity and low immunogenicity [91].

Additionally, attaching PEG onto the surface of nanocarriers facilitated Ganetespib entrapment, as proven by the high encapsulation efficiency of 93.3%. On the other hand, the calculated negative zeta potential (−12.5 mV) and size (228.8 nm) provided the nanocarriers with efficient blood circulation and increased absorption while also increasing cellular uptake for enhanced pharmacological effect. PEGylated nanocarriers represent exceptional nanosystems with great delivery significance [92].

mPEG shows excellent biodegradability and biocompatibility, which assists in targeted delivery of Doxorubicin via entrapment in HPMA nanocarriers, the combination of which resulted in low immunogenicity and a rather wide size distribution of 0.384 (PDI). PEG contributed to the loaded API’s mass being as high as 5.604% of the total delivery system to a small size (46.86 nm), which allowed the nanoparticles to easily pass through the vessels and reach the desired destination, thus providing successful targeting and release regulation [128].

As far as PEGylated nanoparticles are concerned, PEGylation (using PEG-PLGA copolymers) provides water solubility and low toxicity, along with evasion from the immune system response. Using the hydrophilic characteristics to reduce recognition by the body’s defense mechanisms, PEGylation achieves longer-lasting in vivo stability and circulation. PEGylated nanocarriers adjoined with GRGDS assisted in drug delivery by controlling the release of Curcumin into the target organ while also minimizing the possibility of creating side effects and reducing the pharmacological effect [2].

Using PEG as a shielding agent has been researched for longer-lasting circulation in body tissues, avoidance by the biomolecules that protect the body, and better overall evasion of clearance by the immune system. PEG adds biocompatibility, flexibility, aqueous solubility and low toxicity. The obtained physicochemical properties of PEGylated PMMA nanoparticles included a size of 261.2 nm, a size polydispersity index of 0.07, and a zeta potential value of −18.99 mV, indicating that PEGylation created a versatile and uniform environment for stable encapsulation and delivery with a low size distribution [129].

In another study, researchers focused on Paclitaxel, which presents low water solubility, a barrier to enhanced pharmacological properties. In order to ameliorate its effect, administration proceeded via its capture inside PEGylated PAP nanoparticles. mPEG provides stability when it is chemically bound onto nanoparticles and secures the functionality of drugs by contributing physicochemically as a coating agent for enhanced cellular uptake and in vivo circulation. The size of 110.3 nm and the encapsulation efficiency percentage of 42.6% indicated that the PEGylated nanoparticles could contribute to safe delivery of the drugs with steady flow inside the blood vessels [130].

Nanoparticles with PEG attached on the surface in the form of NH_2_-PEG_5000_-NOTA copolymers were developed for application in kidney injury due to their increased safety as encapsulation vehicles. PEG provided hydrophilicity and improved the flow of the nanosystem in the bloodstream by creating a coating domain that ensured reduced recognition by the immune system, as well as enhancing water solubility in order to assist in the transport of APIs. Despite drawbacks like hepatic retention being documented, PEGylation has the potential to be used in drug delivery in order to aid physicochemically for more stable transport [131].

On the other hand, in order to enhance the pharmacological effect of Letrozole, PEGylation of Protamine nanoparticles was studied, with results showing a size of 258 nm and a polydispersity index of 0.114. The nanosystem presented potential when it comes to drug delivery, with PEG being the main factor due to its high toxicity against the targeted cells, as well as its ability to improve the cellular uptake. PEGylation led to enhanced bioavailability via prolonged circulation in vivo, apart from low toxicity to the body itself (IC_50_ of 50 μM) [132].

Finally, PEG_6000_’s low immunogenicity and ability to bind onto ZnO-based nanoparticles for advanced pharmacokinetic and pharmacodynamic potential was proven to boost the life expectancy of the nanosystem and its circulation in vivo, as well as reducing recognition by the immune system, indicating safer encapsulation and delivery. The calculated total mass of the loaded drug was 93% over the non-loaded carrier, showing high pharmacological effect. The only disadvantage is that the temperature needs to be regulated in order to avoid the decomposition of the PEG coating and the destabilization of drug release [133].

## 4. Overview of PEG Functionality

Polyethylene glycol is a multifunctional tool within the scope of drug delivery, serving many roles in both solid dosage forms and nanocarriers due to its physicochemical characteristics. First, it acts as a solubilizer, improving the dissolution profile and bioavailability of poorly water-soluble APIs through molecular dispersion and hydrogen bonding. As a plasticizer and a binding agent, PEG ameliorates the mechanical strength of delivery systems and adds flexibility, which allows these systems to navigate with ease within the human body and regulate delivery. Moreover, its hydrophilic and non-ionic nature minimizes protein adsorption and RES recognition while prolonging the in vivo circulation time. Its ability to act as a conjugation agent in nanocarriers confers higher permeability and retention, as well as reduced aggregation. Finally, as a pore former and release modifier, PEG gives systems precise control over drug diffusion and release kinetics, and at the same time, its amphiphilic structures promote self-assembly and compatibility with both hydrophobic and hydrophilic pharmaceutical ingredients [1,2,3,4,5,6].

PEGylation is a formulation strategy to improve nanocarriers’ physicochemical and biological stability by prolonging their circulation time, but it also influences immune responses. Namely, the repeated administration of PEGylated nanomedicines or conjugates (polymer–drug or polymer–protein conjugates) can induce the production of anti-PEG antibodies. Consequently, the production of anti-PEG antibodies is responsible for the accelerated blood clearance (ABC) phenomenon [134,135,136]. For this reason, the opsonization phenomenon and removal by the mononuclear phagocyte system can alter circulation time, drug bioavailability, and immune activation. According to the recent literature, pre-existing anti-PEG antibodies are found in healthy individuals in a significant proportion of the population. Considering these clinical observations, several other polymers which are biocompatible and/or biodegradable and able to exhibit “stealth” properties have appeared as alternative biopolymers for use in nanocarriers. Poly(2-oxazoline) is one of the most attractive synthetic and marketed polymers available for conferring “stealth” properties [137].

Molecular weight, chain length, and end-group chemistry are features that affect PEG’s solubility, hygroscopicity, compatibility, and miscibility with other excipients and APIs in drug formulations. Therefore, these characteristics influence the drug dissolution, release rate, mechanical behavior, and stability of the product. Low-MW PEGs are in liquid form and act as solubilizers or wetting agents, enhancing drug wettability and consequently dissolution rate. Also, PEG chain mobility increases with low MW, so low-MW PEGs can act as plasticizers in solid dispersions and film coatings, improving tablet flexibility and preventing brittleness. On the other hand, high-MW PEGs are crystalline solids and can function as matrix formers, reducing the dissolution rate, and can be used in sustained-release systems. The combination of PEGs with different MWs can tailor drug release kinetics [138]. Furthermore, PEGs with reactive end groups or those integrated with copolymerization exhibit tunable miscibility and improved physical stability in solid dosage forms [139].

PEG chains form a hydrophilic shield around the nanoparticle surface, which prevents complement deposition and the formation of the membrane attack complex, leading to complement activation. In other words, the cellular interactions and the intracellular pathways change significantly. The PEG brush structure also leads to avoiding protein absorption onto the nanocarriers’ surface, i.e., avoiding opsonin binding. The limited protein (albumin and other serum proteins) binding cannot be detected from pattern recognition receptors (PRRs), leading to minimum macrophage uptake. The limited receptor-mediated uptake influences endocytosis efficiency and modulates endosomal escape. Intracellular trafficking is also affected, since the endosomal and recycling biological pathways change due to the change in the ADMET profile of the encapsulated active pharmaceutical ingredients. Molecular weight, density, macromolecular architecture, and conformation are the main factors that affect the mechanism of how PEG coatings interfere with complement activation, opsonin binding, and macrophage uptake [140].

Given the potential drawback of PEGylated nanocarriers accelerating blood clearance, alternative strategies have been developed, such as utilizing red blood cell membranes to achieve immune evasion. Specifically, recent studies designed and developed red blood cell membrane-derived delivery platforms that enhance the prolonged circulation of anticancer treatment strategies and overcome the observed ABC phenomenon induced by PEGylation [141,142].

In recent years, especially after the design and development of COVID-19 vaccines, PEGylation has been the main surface coating of lipid nanoparticles and protein-based immunotherapies. As mentioned above, PEGylated nanoparticles exhibit good steric stabilization and physicochemical stability due to the presence of hydrophilic polymeric chains on the surface of the nanoparticles. The presence of PEG chains is also responsible for avoiding the absorption of albumin and other serum proteins. For this reason, the supramolecular aggregates of nanoparticles and plasma proteins are not detected, leading to prolonged circulation time and limited immune recognition and response. Furthermore, these phenomena are responsible for limited antigen presentation, endosomal escape, and endocytosis [140].

On the other hand, we should point out that alternative polymers have already been synthesized to be used for the formulation of nanoparticles with stealth properties. Namely, He et al. (2024) [143] designed and developed mRNA–lipid nanoparticles using poly(2-oxazoline) (POx)- and poly(2-oxazine) (POz)-based polymer–lipid conjugates. The results from biophysical characterization and cytocompatibility studies, as well as studies in mice, showed that these polymers are promising alternative candidates for PEG-free nanoformulations. In the same context are the findings obtained by Holick et al., 2025 [144], who showed the decisive role of such next-generation polymers [143,144]. Additionally, poly(sarcosine) surface modification imparts stealth-like properties and biological stability to conventional liposomes by enhancing circulation time and reducing macrophage recognition [145]. Some years later, a comparative study between PEGylation and polysarcosine surface coatings in block copolymer nanostructures encapsulating anticancer drugs in tumor models showed the improved endocytosis and immunogenic cell death of systems with polysarcosine surface modification [146].

## 5. Conclusions

PEG is an important hydrophilic polymer that exhibits high potential in the development of drug delivery systems, either as a coating agent of nanocarriers or in solid dosage form applications. Concerning its molecular properties, it provides great biodegradability and biocompatibility, along with the ability to be solubilized inside the human body without posing major health hazards, thus leading to a higher ecological footprint and making it more accessible for research. It can also play a catalytic role, acting as a carrier or plasticizer in the form of a copolymer, or it can play a role in the control of hygroscopicity for the manufacturing/administration of solid forms with a modified release profile (prolonged, sustained, or immediate). Moreover, PEG alters the mechanical properties of the final formulation, affecting the lifetime of drugs. PEG’s ability to act as a plasticizer or a pore former enables the design of different and innovative categories of drugs, such as orodispersible, osmotic pump, or coated tablets, aiming at meeting the demands of the pharmaceutical industry that works for the alleviation of human pain and the amelioration of the quality of life.

Concerning its added value as a component of nanocarriers and a conjugation agent, PEG enhances the in vivo circulation of the nanosystem by taking advantage of its physicochemical properties, avoiding clearance by the body’s defense mechanisms. Several PEGylated nanomedicines are already on the market. On the other hand, some recent studies have tried to present new candidates for the design and development of “stealth” nanocarriers with added value in nanotherapeutics.

In conclusion, taking into account the wide range of uses of PEG, we should point out that it is an excipient with many uses both in solid dosage forms and in advanced drug delivery systems. It will play a leading role in the research and development of new APIs in various dosage forms. The efforts to replace it mainly concern nanocarriers. But in any case, a formulation scientist should know the properties of PEG to improve the ADMET profile of a drug.

## Figures and Tables

**Table 1:** Collective data summing up the added value of PEG in the process of drug delivery in solid dosage forms.

Formulation	Characteristics of PEG Utilized	Characteristics of the Formulation	Added Value	Functionality of PEG	References
Self-nanoemulsifying tablets	Molecular weight: 40–400; high transmittance: 94–99%	Hardness: 4.23 kg/cm^2^; friability: 0.29%; disintegration time: 49 s; weight: 700 mg; drug content: 96.42%; dissolution: 84% (15 min)	Improved solubility and oral bioavailability, immediate release, reduced production cost, stability, simplified manufacturing, accurate dosing, and improved patient compliance	Surfactant, cosurfactant, and emulsifier	[29]
Orodispersible tablets	Molecular weight: 6000; Differential Scanning Calorimetry: exothermic peak at 63 °C	Drug solubility: 4.50 mg/mL; weight: 147–152 mg; hardness: 3.12–3.50 kg; thickness: 2.99–3.05 mm; DSC: thermograph at 230 °C; ΔHf: 239 J/g	Increased solubility at lower pH and increased drug release	Hydrophilic carrier	[33]
Orodispersible tablets	High hydrophilicity	Thickness: 3.47–3.89 mm; size: 9.95–10.03 mm; hardness: 4.1–10.2 kg/cm^2^; wetting time: 28–96 s; friability: 0.30%; disintegration time: 18.7 s	Adequate mechanical strength and faster disintegration time	Binding agent; enhances the dissolution of poorly soluble compounds, reduces pill burden, and overcomes swallowing problem	[34]
Coated tablets	Molecular weight: 3350, i.e., 30% *w*/*w* of the formulation	Coating polymer: PVA or HPMC plasticizer/detackifier, PEG or triacetin; API/coating material ratio: 1/6; HPMC-PEG: pH = 6.5; PVC-PEG: pH = 5.8; stability: triacetin better than PEG and HPMC than PVA	Enhanced solubility and reduced degradation	Plasticizer or detackifier	[35]
Cushion-coated pellet systems	Molecular weight: 1500; soft and soluble, with high solubility and low melting temperature (44–48 °C)	Thickness: 30 μm; disintegration time: 8–9.7 min	Administration of multiple-unit dosage forms	Improved mechanical strength, rapid disintegration, integrity of pellets, and compaction	[36]
Modified release, 3D-printed	Molecular weight: 700; photoreactive and hydrophilic	Size: 10 mm; thickness: 3 mm; weight change: 2.02%; friability: 0.96%; in vitro drug release after 24 h: 79%	Sustained release, increased bioavailability, and C_max_ of 30.1 μg/mL	PEG-DA 700 photopolymer PEG 400 excipient; improvement in dissolution and release	[37]
Tablets with modified release	Molecular weight: 8000; melting point: 60 °C	Size: 7 mm; oval tablets; weight: 615 mg; thickness: 6.8 mm; drug release kinetics: Korsmeyer–Peppas	Decreased frequency, better patient compliance, and fewer side effects	Controlled hygroscopicity and retarded drug release	[38]
Tablets with modified release	Molecular weight: 1500–8000; hygroscopic semi-crystalline character, with good plasticizer properties	Reduced drug crystallinity; thickness: 4.02–4.16 mm; size: 11.9–12.1 mm; weight: 492.1–506.3 mg; disintegration time: 10–160 s	Immediate release, optimal time of disintegration, and direct absorption by GIT	Carrier and copolymer	[39]
Capsules with special characteristics	Molecular weight: 20,000; hydrophilic, flexible, resistant to immunological recognition, and biocompatible	PLA-PEG ratio: 6/4; size: 6 μm; release ratio: 66%	Clinical safety and no cytotoxicity	Shell material and carrier	[40]
Capsules with targeted administration	PEG methyl ethyl acrylate; molecular weight: 480; PEG dimethyl acrylate for the fabrication of LPEG capsules; 8-arm PEG acrylate for the fabrication of MPEG capsules	Soft capsules, size of several micrometers, and negative zeta potential (presence of silicon oxide in the inner layer)	Biocompatibility, enhanced cellular uptake, low cell association, and improved delivery efficacy	Reduction in non-specific interactions, circumvention of biological carriers, and modification of nanocarriers	[41]
Enteric capsules	PEG Monomethyl Ether; molecular weight: 2000; part of copolymer PLGA-Hyd-PEG	Size: 130–140 nm; polydispersity index: <0.2	Glucose reduction of up to 35% for a period of time of up to 10 h	Fabrication of pH-sensitive core and conversion of nanoparticle surface to hydrophobic	[42]
Enteric capsules	Molecular weight: 4000	Moisture after the packaging process: 13.6%; gelation temperature: 34 °C; gelation time: 19 s; smooth surface; stable in the stomach for 120 min	Fabrication of enteric capsules without the coating step	Filler; reduces the moisture of capsules, eliminates porosity, and increases stability in the stomach	[43]

**Table 2:** Collective data summing up the added value of PEG in the process of drug delivery in nanoformulations.

Formulation	Characteristics of PEG Utilized	Characteristics of the Nanocarrier	Added Value	Functionality of PEG	References
Liposomes	Hydrophilicity; non-ionic, with high density, cytocompatibility, non-immunogenicity, and high permeability; molecular weight: 2000–8000	Unilamellar, electronically neutral, and uniform; size: 90–240 nm; zeta potential: −0.1–(−61) mV; polydispersity index: 0.14–0.26; EE: 43–98%; LE: 8.5–8.8%	T-cell independence, larger protein adoption, improved in vivo stability, hemocompatibility, and insignificant macrophage uptake	Increased stability, decreased toxicity, and increased biodistribution, antigenicity, and biocompatibility	[4,44,45,46,47,48,49,50,51,52]
Micelles	Hydrophilicity, molar mass dispersity, flexible structure, and low cytotoxicity; molecular weight: 1000–5000	Anionic charge, amphiphilicity, and feasible manufacturing; size: 17–350 nm; zeta potential: −27–(−4.5) mV; polydispersity index: 0.11–0.47; EE: 41–99%; LE: 3–20%	Targeting regulation, prolonged circulation, efficient accumulation at the tumor site, reduced toxicity, and avoidance of macrophage clearance	Biocompatibility, biodegradation, temperature sensitivity, high water solubility, decreased opsonization by the RES system, and IC_50_ of 7 ± 14 μg/mL	[53,54,55,56,57,58,59,60,61,62]
Polymersomes	Hydrophilicity, high surface density, and large chain length; molecular weight: 750–5000	Amphiphilicity, core–shell structure, thicker membranes, and high loading capacity; size: 33–265 nm; zeta potential: −20–(−0.3) mV; polydispersity index: 0.13–0.26; EE: 68–98%; LE: 0.9–52%	Higher retention time, deterrence of membrane opsonization, and enhanced systemic half-life in blood	Biocompatibility, decreased toxicity, reduced plasma protein adsorption, and resistance to cellular adhesion	[63,64,65,66,67,68,69,70,71,72]
Inorganic nanoparticles	High surface density, hydrophilicity, low cytotoxicity, and non-immunogenicity; molecular weight: 400–8000	Superparamagnetism, colloidal stability, and reduced inflammatory damage; size: 6.8–650 nm; zeta potential: −36–0.6 mV; polydispersity index: 0.09–0.16; EE: 68–90%	Enhanced blood circulation time, and reduced opsonization and subsequent clearance by the phagocytosis system	Reduced toxicity, high water solubility, moisture retention, filtration and sterilization effects, biosafety, and IC_50_ of 46 ± 50 μg/mL	[73,74,75,76,77,78,79,80,81,82]
Niosomes	Hydrophilicity, cytotoxicity on cancer cells, and flexibility; molecular weight: 600–6000	Non-ionic surfactant, spherical; size: 117–273 nm; zeta potential: −43–(−1.7) mV; polydispersity index: 0.08–0.54; EE: 72–96%; LE: 1.2–17%	Enhanced cellular uptake, improved curative properties, limited RES system capture, increased half-life, improved storage stability, and reduced side effects	Biocompatibility, protective coating agent, protection against degradation, increased cell viability, and IC_50_ of 0.43 ± 88 μg/mL	[83,84,85,86,87,88,89,90,91,92]

## Data Availability

No new data were created or analyzed in this study.

## Abbreviations

PEG	Polyethylene Glycol
API	Active pharmaceutical ingredient
PRISMA	Preferred Reporting Items for Systematic Reviews
C_max_	Maximum Concentration
AUC	Area Under Curve
DCT	Directly compressed tablets
3D	Three-dimensional
DSC	Differential Scanning Calorimetry
Type 2DM	Type 2 Diabetes Mellitus
PVP	Polyvinylpyrrolidone
HPMC	Hydroxypropyl Methylcellulose
PVA	Polyvinyl Alcohol
T_max_	Time to Maximum Concentration
P-gp	P-glycoprotein
PEG-DA	Polyethylene glycol diacrylate
UV	Ultraviolet radiation
HIV	Human Immunodeficiency Virus
Tg	Glass Transition Temperature
MW	Molecular weight
DTZ	Diltiazem Hydrochloride
PEO	Polyethylene Oxide
NMR	Nuclear Magnetic Resonance
GIT	Gastrointestinal tract
POL188/P188	Poloxamer 188
ALA	Alpha Lipoic Acid
mRNA	Messenger Ribonucleic Acid
PLA	Polylactic Acid
SO_2_	Sulfur Dioxide
SN-38	7-Ethyl-10-hydroxycamptothecin
PCL	Polycaprolactone
PLGA	Poly(lactic-co-glycolic acid)
ABZ-SO	Albendazole Sulfoxide
DSPE	1,2-Distearoyl-sn-glycero-3-phosphoethanolamine
DPPC	Dipalmitoylphosphatidylcholine
PDI	Polydispersity index
DMF	Dimethyl Formamide
IC_50_	Half-maximal Inhibitory Concentration
PdLLA	Poly-D,L-Lactic Acid
NP	Nanoparticle
Span 60	Sorbitan Monostearate
Tween 60	Polyoxyethylene Sorbitan Monostearate
HPMA	Polymer N-(2-hydroxypropyl) Methacrylamide
GRGDS	Glycyl-arginyl-glycyl-aspartyl-serine
PMMA	Poly(methyl methacrylate)
ZnO	Zinc Oxide
